# Editorial: Bioactive metabolites in traditional medicine: dual pathways to metabolic health

**DOI:** 10.3389/fphar.2025.1729667

**Published:** 2025-11-06

**Authors:** Wenlong Sun

**Affiliations:** School of Life Sciences and Medicine, Shandong University of Technology, Zibo, Shandong, China

**Keywords:** natural products, metabolic diseases, gut microbiota, traditional medicine, dual pathways

Traditional medicines and natural products have long been regarded as promising resources for combating metabolic diseases. These products and their active metabolites interface with the body through various pathways, primarily blood-entry and non-blood-entry routes. Some small molecules in natural drugs, such as flavonoids, can be absorbed in the intestinal tract and enter the circulatory system, while polysaccharides, saponins and other components are difficult to be absorbed directly, but can be played by intestinal microorganisms. Extensive investigations have been conducted to comprehensively elucidating their therapeutic potential and practical applications. However, their “multi-component, multi-target, multi-pathway” mode of action presents substantial challenges, underscoring the need for further in-depth research.

The pathway blood-entry is the active substance enter the blood circulation through absorption, and exert its effect by regulating the whole body signal pathway or directly acting on the target organ. The research mostly involves pharmacokinetics, transcriptomics, metabolomics, and molecular docking to investigate the pharmacological actions and underlying mechanisms of natural medicinal compounds. At present, this topic includes three studies on this approach.

Clarifying the active ingredients of natural products, pharmacological effects and mechanisms of action holds the key to achieving clinical transformation. Naz Qaisrani et al. conducted a systematic investigation on the essential oil of *Pinus roxburghii* (*Integrative in silico and in vitro analysis of P. roxburghii essential oil: unveiling its antioxidant, antidiabetic, and antiglycation potential*), identifying β-pinene and caryophyllene oxide as the primary active components through GC-MS analysis. Molecular docking experiments demonstrated that α-myricene could stably bind to the active pocket of α-glucosidase (ΔG = −7.7 kJ mol^-1^). *In vitro* experiments further verified the substantial inhibitory effect of the essential oil on α-glucosidase, effectively curbing advanced glycation end products formation.

Shao et al. investigated the pharmacokinetic characteristics of *Gastrodia elata* (GE) extract within cerebral ischemia models, addressing a lacuna in the research on the *in vivo* processes of traditional medicine under pathological conditions (*Pharmacokinetics and brain tissue distribution of* GE *extract in normal and cerebral ischemic rats: a comparative study*). The research revealed that although the absorption efficiency of GE active components (e.g., gastrodin and parishin A-E) decreased within the gastrointestinal tract, their concentration in the brain increased 1.8 to 12.9 times compared to normal rats due to compromised blood-brain barrier (BBB) integrity. Additionally, the drug retention time in the brain was prolonged from 0.6 h to 0.88 h (T_1_/_2_). The study identified nine metabolites of GE, in which 4 were capable of entering the brain to exert effects, providing a basis for the development of GE prodrugs.

Liu et al. established a fundamental precedent for multi-omics-driven mechanistic research in traditional Mongolian medicine by investigating Saorilao-4 decoction (SRL) in bleomycin-induced pulmonary fibrosis (PF) rats (*Unveiling the therapeutic mechanisms of Saorilao-4 decoction in pulmonary fibrosis through metabolomics and transcriptomics*). Through integrated transcriptomics and metabolomics, Liu et al. identified that SRL targets AMPK and PPAR, signaling pathways by regulating genes such as *Scd*, *Fads2*, and *Cpt1a*. This regulation restores dysregulated metabolic pathways that are critical to the progression of PF. Histopathological and biochemical analyses further verified SRL’s capacity to reduce lung collagen deposition and inflammatory cytokines levels (IL-1β, IL-6), providing the evidence for SRL’s anti-fibrotic effects.

Polysaccharides and other active compounds are difficult to be absorbed directly by the intestinal tract. Instead, they function as prebiotics or microbial modulators. These compounds influence systemic metabolism and target organ functions indirectly by altering gut microbiota composition and regulating microbial metabolites. Current research primarily involves gut microbiota sequencing, detection of microbial metabolites, and correlation analysis of the “microbiota-metabolite-host” axis. In this topic, Liao et al. and Li et al. investigated the mechanisms by which traditional Chinese medicine formulas and Guanxin Qiwei Dropping Pills (GXQW) exert their effects through gut microbiota.

Liao et al. (*The mechanism of Guanxin Qiwei dropping pills target Dubosiella to improve atherosclerosis*) integrated gut microbiota research with traditional Chinese medicine pharmacology by investigating GXQW in high-fat diet-induced atherosclerotic ApoE^−/−^ mice. Liao et al. found that GXQW effectively regulates the gut microbiota structure in ApoE^−/−^ mice induced by a high-fat diet, reducing the abundance of Firmicutes and particularly suppressing the overgrowth of the genus *Dubosiella*. Meanwhile, GXQW effectively reduces serum p-cresol sulfate (PCS) levels, while decreasing vascular endothelial oxidative stress and inhibiting NF-κB pathway activation, thereby reducing aortic plaque coverage and lipid deposition. Correlation analysis further revealed a positive association between *Dubosiella* and PCS, identifying the “*Dubosiella*-PCS axis” as a key target of GXQW. Liao et al. not only validated GXQW’s anti-atherosclerotic effects but also extended the “gut microbiota-metabolite axis” concept to traditional Chinese medicine, demonstrating how traditional formulas can target microbial dysbiosis to treat metabolic diseases.

Li et al. investigated OJPS (*Ophiopogon japonicus* polysaccharide) and discovered that it acts as a prebiotic to stimulate the proliferation of beneficial gut bacteria such as *Lactobacillus* and *Bifidobacterium* (*Advances in the study of O. japonicus polysaccharides: structural characterization, bioactivity and gut microbiota modulation regulation*). These probiotics ferment OJPS to produce short-chain fatty acids (SCFAs), which stimulate intestinal L-cells to secrete GLP-1, thereby promoting insulin secretion. Simultaneously, they inhibit hepatic gluconeogenesis through GPR41/GPR43 receptors, ultimately reducing blood glucose levels in diabetic mice. Moreover, Li et al. discovered that the ultrasonically assisted extraction of OJPS, with its lower molecular weight, enables facile fermentation by gut microbiota, resulting in 30% higher hypoglycemic activity compared to traditional hot water extraction.

Notably, the efficacy of natural medicines often stems from the synergistic effects of both blood-entry and non-blood-entry. According to a review included in this topic, flavonoids, which are blood-entry, and polysaccharides, which are non-blood-entry, in astragalus are involved in the anti-tumor effect.

Liu et al. further broadened the application scope of traditional Chinese medicine in complex diseases by focusing on the anti-tumor potential of *Astragalus membranaceus*, and its active metabolites (*Metabolites of Astragalus membranaceus and their pro-apoptotic and cytotoxic activities: insights into targeted metabolic pathways*). The review systematically identified three core active components—polysaccharides (APS), saponins, and flavonoids, and clarified their multi-target anti-tumor mechanisms. This research not only verified the potential of astragalus as an adjuvant anti-tumor drug, but also suggested that future research could further explore the synergistic mechanisms of natural drugs’ blood-entry and non-blood-entry pathways, thereby clarifying their target sites and advancing their development and clinical applications.

This thematic compilation conducts systematic review of the recent research progress in the utilization of medicinal plants, their extracts, and metabolites for the prevention and treatment of metabolic diseases. Aims to identify and characterize bioactive metabolites, clarify their mechanisms of action, and explore their interactions with metabolic pathways and gut microbiota. Through the investigation of dual pathways to metabolic health, novel preventive and therapeutic strategies for improving metabolic health.

